# Functional and Structural Properties of Cytoplasmic Tropomyosin Isoforms Tpm1.8 and Tpm1.9

**DOI:** 10.3390/ijms25136873

**Published:** 2024-06-22

**Authors:** Ksenia K. Lapshina, Victoria V. Nefedova, Salavat R. Nabiev, Svetlana G. Roman, Daniil V. Shchepkin, Galina V. Kopylova, Anastasia M. Kochurova, Evgenia A. Beldiia, Sergey Y. Kleymenov, Dmitrii I. Levitsky, Alexander M. Matyushenko

**Affiliations:** 1 Research Centre of Biotechnology, Russian Academy of Sciences, 119071 Moscow, Russia; lapshina.2003@gmail.com (K.K.L.); victoria.v.nefedova@mail.ru (V.V.N.); svetabaj@gmail.com (S.G.R.); s.yu.kleymenov@gmail.com (S.Y.K.); levitsky@inbi.ras.ru (D.I.L.); 2 Department of Biophysics, Faculty of Physics, Lomonosov Moscow State University, 119234 Moscow, Russia; 3 Institute of Immunology and Physiology, Ural Branch of Russian Academy of Sciences, 620049 Yekaterinburg, Russia; salavatik2003@gmail.com (S.R.N.); cmybp@mail.ru (D.V.S.); g_rodionova@mail.ru (G.V.K.); kochurova.a.m@mail.ru (A.M.K.); zgupkabeldya@gmail.com (E.A.B.); 4 Koltzov Institute of Developmental Biology, Russian Academy of Sciences, 119334 Moscow, Russia

**Keywords:** actin filaments, cytoplasmic isoforms of tropomyosin, actin cytoskeleton dynamics, actin-associated proteins, optical trap, differential scanning calorimetry

## Abstract

The actin cytoskeleton is one of the most important players in cell motility, adhesion, division, and functioning. The regulation of specific microfilament formation largely determines cellular functions. The main actin-binding protein in animal cells is tropomyosin (Tpm). The unique structural and functional diversity of microfilaments is achieved through the diversity of Tpm isoforms. In our work, we studied the properties of the cytoplasmic isoforms Tpm1.8 and Tpm1.9. The results showed that these isoforms are highly thermostable and differ in the stability of their central and *C*-terminal fragments. The properties of these isoforms were largely determined by the 6th exons. Thus, the strength of the end-to-end interactions, as well as the affinity of the Tpm molecule for F-actin, differed between the Tpm1.8 and Tpm1.9 isoforms. They were determined by whether an alternative internal exon, 6a or 6b, was included in the Tpm isoform structure. The strong interactions of the Tpm1.8 and Tpm1.9 isoforms with F-actin led to the formation of rigid actin filaments, the stiffness of which was measured using an optical trap. It is quite possible that the structural and functional features of the Tpm isoforms largely determine the appearance of these isoforms in the rigid actin structures of the cell cortex.

## 1. Introduction

The actin cytoskeleton is one of the most important parts of a living cell, ensuring its function and regulation of many processes: cell adhesion, vesicle transport, cytokinesis, endocytosis, morphogenesis, etc. [1]. Microfilaments built from F-actin penetrate the villi and form a strong network at the leading edge of moving cells [2]. The implementation of such diverse and complex functions would be impossible without the actin partner protein, tropomyosin (Tpm) [3]. Tpm forms an extended cable that interacts with the surface of the actin filament. By wrapping actin, Tpm regulates the stiffness, flexibility, and stability of actin filaments [4]. Moreover, it serves as a kind of a backbone for the assembly of complexes with other actin-binding proteins [5]. In a living cell that is not in the stage of active division or movement, most of the actin filaments are decorated with Tpm [6].

Tpm is a left-handed coiled-coil protein consisting of two right-handed α-helices. The coiled-coil structural motif is quite common; about 3–5% of all amino acids in proteins are able to form it [7]. The tertiary structure of this motif is largely determined by features of the primary sequence, which is divided into seven-membered repeat heptads [8]. For the stability of the coiled-coil structure, the first and fourth positions (**a** and **d**) in the heptad are of great importance since they form the hydrophobic core of the molecule [9,10]. The fifth and seventh positions in the heptad are also significant. They additionally stabilize the structure and determine the parallel and antiparallel formation of the superhelix [11]. For the Tpm structure, it was shown that the helices with the highest content of hydrophobic residues in the **a** and **d** heptad positions have the greatest stability [10]. The preference in position **a** is for β-branched amino acids (Ile, Val), while in **d**—for unbranched or γ-branched residues (Leu) [9]. To form interhelical electrostatic interactions, the residues at positions **e** and **g** must be charged. Bonds are formed according to the rule i → i’ + 5, when residue **g** of one helix forms an ion pair with residue **e** of the next heptad of the second helix [11].

Tpm has many functions in the cell. Functions directly related to the regulation of actin filaments include stabilization of the “minus” end of the actin filament [12], protecting it from the rupture and depolymerization by other proteins [13,14,15], and formation of a complex with Arp2/3 [16,17]. Also, Tpm plays a key role in regulating the interaction between actin and myosin [18]. The functional diversity is provided by a large number of Tpm isoforms obtained by the alternative splicing of four genes [19]. The features of the exonic structure, as well as the isoform composition of human, mouse, and rat forms of Tpm, were taken into account in the current nomenclature [20].

The Tpm1.8 and Tpm1.9 isoforms (Tm5a and Tm5b in the old nomenclature, respectively) are the products of the selection of the alternative promoter (1b) and alternative splicing exons 6 and 9 of the *TPM1* gene [20]. They belong to the class of short non-muscle isoforms (LMW) containing 247 amino acid residues. In the LMW isoforms, the first exon (exon 1b, encoding residues 1–43) replaces the first two exons (exons 1a and 2, encoding residues 1–80) of the long isoform (HMW). The two alternatively spliced exons are exons 6 and 9 [20]. The actin-binding properties are thought to be determined primarily by the two terminal regions encoded by exons 1 and 9 [21]. Tpm1.8 and Tpm1.9 extend to the cell periphery in fibroblasts and epithelial cells. They are most abundant in the kidney, liver, lung, and spleen and were not detected in the brain [22].

The function of Tpm1.9 in erythrocytes has been studied in detail [23,24]. Short F-actins coated by Tpm1.9 and Tpm3.1 are key entities in the erythrocyte membrane scaffold [25,26]. The absence of Tpm3.1 in erythrocytes (obtained by the deletion of exon 9d of *TPM3* gene) leads to a compensatory increase in Tpm1.9 in the membranous skeleton [27,28]. However, exclusive expression of Tpm1.9 results in red blood cell F-actins becoming abnormally resistant to depolymerization [23]. This correlates well with Tpm1.9’s more than 2-fold higher affinity for F-actin and increased efficiency of reducing F-actin depolymerization in vitro [29]. Moreover, the Tpm1.9 interaction with Tmod1 is stronger than that with Tpm3.1 [24], suggesting that Tpm1.9 is more efficient compared to Tpm3.1 in anchoring Tmod1 to the pointed end, providing further inhibition of F-actin depolymerization.

For Tpm1.8, the kinetics of binding to actin filaments have been studied in detail [30]. It was shown that the elongation of the Tpm1.8 polymer to the barbed end of the actin filament occurred faster than to the pointed end with 1.85 ratio between the two rate constants. Also, according to experimental data, the nucleation of the second Tpm chain on the surface of the actin filament turned out to begin independently of the first chain [30].

In our work, we studied the structural and functional features of the cytoplasmic isoforms of tropomyosin Tpm1.8 and Tpm1.9. Although it is conceivable that the fine features of the primary/secondary/tertiary structure underlie the peculiarities in the functioning of different isoforms, there are few studies examining this for human Tpm1.8 and Tpm1.9 isoforms. In the present work, we tried to fill this gap and tested whether alanine-serine *N*-terminal modification (AS), mimicking *N*-terminal acetylation of Tpm, affects the properties of Tpm1.8 and Tpm1.9.

## 2. Results

### 2.1. Properties of Isolated Tpm Molecules

At first, we estimated the stability of the Tpm molecules using differential scanning calorimetry (DSC) and the strength of the end-to-end interactions between adjacent Tpm molecules by viscosity measurements.

#### 2.1.1. Differential Scanning Calorimetry Experiments

**Figure 1:**
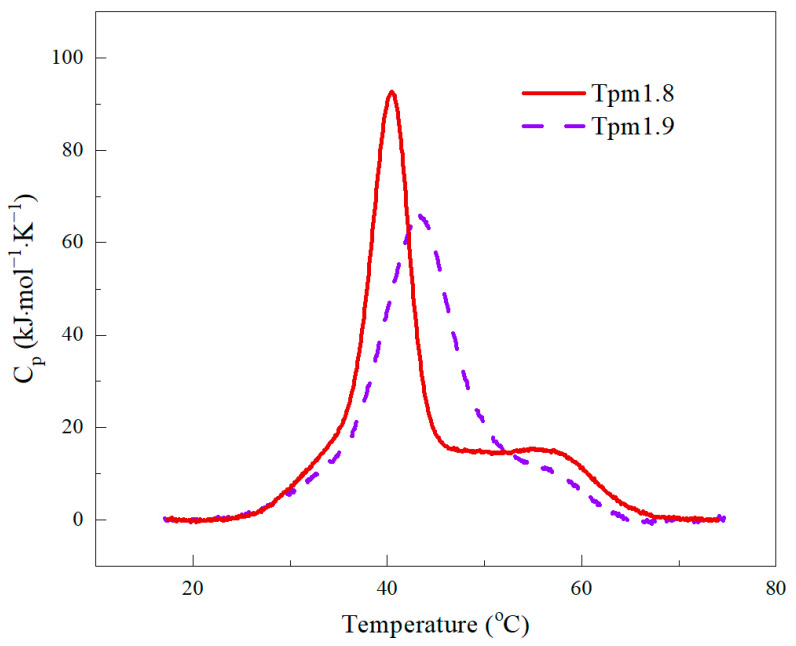
The DSC profiles of the excess heat capacity obtained for Tpm1.8 and Tpm1.9.

**Figure 2:**
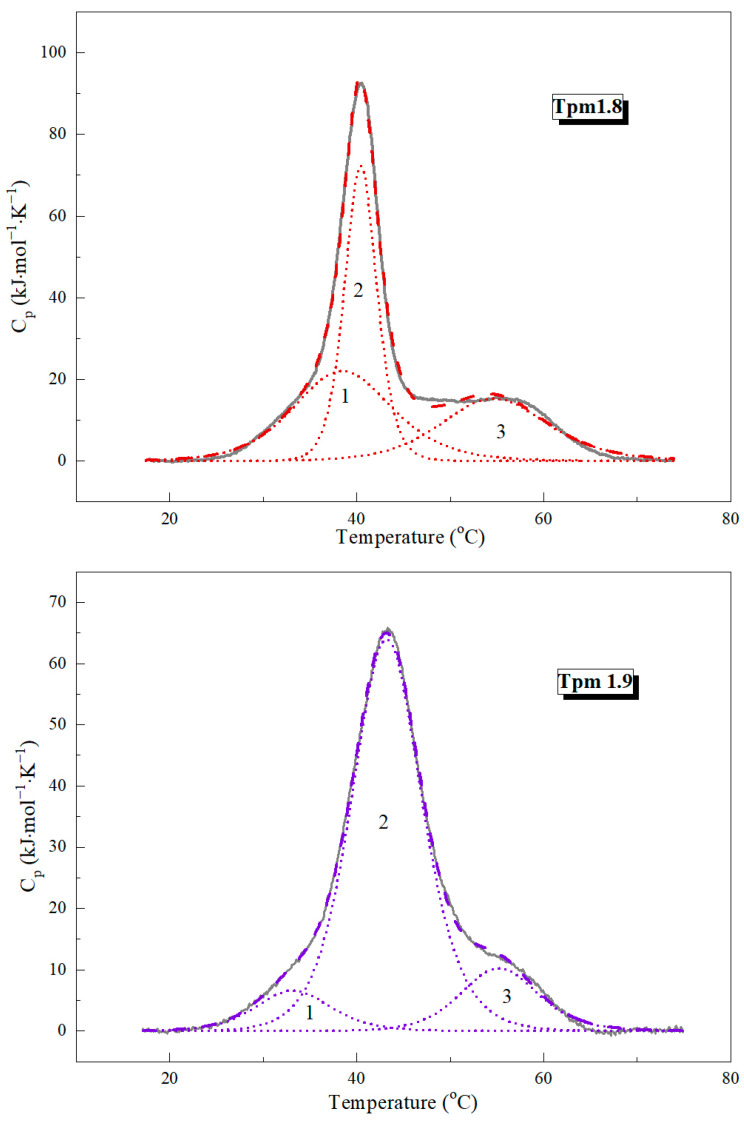
The deconvolution analysis of the heat absorption curves of Tpm1.8 and Tpm1.9. The solid curves represent the experimental profiles after subtraction of the instrumental and chemical baselines, and the dotted lines represent the individual thermal transitions (calorimetric domains 1–3) obtained by fitting to the non-two-state model [19].

DSC was applied to study the thermal stability of Tpm1.8 and Tpm1.9. The heat absorption curves of the Tpm molecules are presented in Figure 1. The results of deconvolution analysis are shown in Figure 2. The thermodynamic parameters of the DSC analysis are given in Table 1.

The major thermal transition for both Tpm1.8 and Tpm1.9 occurred in the temperature range from 39 to 45 °C. Also, two shoulders are present before and after the main peak in the DSC curves (Figure 1). A deconvolution analysis was performed to obtain more detailed information on the thermal stability of the Tpm isoforms. Three calorimetric domains were identified for each Tpm isoform (Figure 2; Table 1). The temperature of the maximum thermal transition (T_m_) for domain 3 (which usually corresponds to the melting of the *N*-terminal part of the Tpm molecule) was similar for Tpm1.8 and Tpm1.9. The major transition corresponds to calorimetric domain 2. This domain had a higher melting temperature for Tpm1.9 (by about 3 degrees) than for Tpm1.8 (Table 1). Calorimetric domain 1 showed the most pronounced differences in the values of thermodynamic parameters. T_m_ values differed by more than 5 °C between isoforms (Table 1). The domain enthalpy obtained for Tpm1.9 was much lower than that for Tpm1.8 (Figure 2; Table 1). Notably, the overall enthalpy was approximately the same for Tpm1.8 and Tpm1.9, while the transition enthalpy varied within the calorimetric domains. Our results indicate the differences in the distribution of stability throughout the whole molecule for these Tpm isoforms. As for AS-Tpm isoforms, the DSC profiles were identical to those of the non-modified isoforms.

#### 2.1.2. Viscosimetry of Tpm Solutions

**Figure 3:**
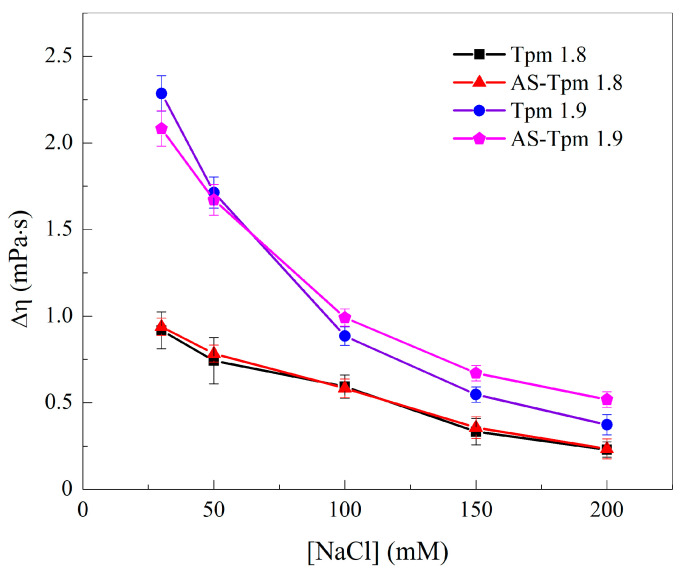
The effect of the ionic strength on the excess viscosity of various Tpm isoform solutions. Experiments were carried out in the 30 mM Hepes buffer, pH 7.3 at a Tpm concentration of 1 mg/mL. Δη—viscosity measured in mPa·s.

The ability of adjacent Tpm molecules to form end-to-end interactions was assessed by measuring the viscosity η of the Tpm solutions. The viscosities of the Tpm1.9 and AS-Tpm1.9 solutions were highly dependent on the ionic strength; the excess (compared to the value for a buffer without additions) viscosity, Δη, decreased from approximately 2.25 mPa·s for 30 mM NaCl close to 0.5 mPa·s for 200 mM NaCl (Figure 3). A more gradual decrease in the Δη([NaCl]) dependence was observed for the Tpm1.8- and AS-Tpm1.8 samples. The range of viscosity changes was around 0.7 mPa·s, and the curves almost coincided in these cases. At the same time, AS-extension slightly increased the viscosity of the Tpm1.9 isoform at [NaCl] = 100–200 mM. It is noteworthy that the Δη value for the Tpm1.9 isoform was almost 2.5 times higher than that for the Tpm1.8 isoform at a low ionic strength of 30 mM NaCl. However, the difference in the strengths of the end-to-end interactions decreased as the ionic strength increased.

### 2.2. Affinity of Tpm to F-Actin and Stability of Its Complexes

The parameters of the Tpm interaction with F-actin were assessed in this section of the study. The affinity of the Tpm molecules was estimated using a widespread co-sedimentation analysis. The stability of the Tpm–F-actin complexes was measured using light scattering experiments, which allowed the dissociation of Tpm from the actin filament surface to be monitored.

#### 2.2.1. Co-Sedimentation Assay

**Figure 4:**
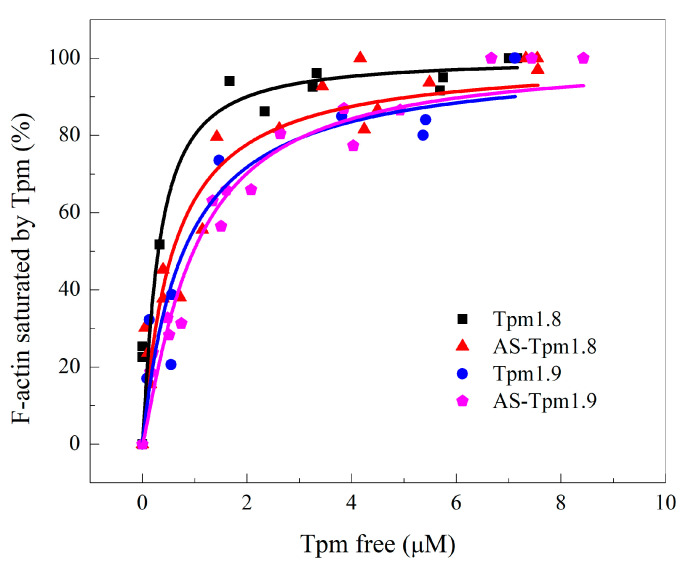
The affinity of different Tpm isoforms for F-actin. F-actin bound Tpm fractions were plotted against the concentration of free Tpm found in the supernatant.

The affinity of all Tpm samples was high, as can be judged by the shape of the curve of actin saturation with Tpm (Figure 4). The full saturation was achieved very quickly, with free Tpm concentrations of around 2 µM for all samples. The K_50%_ values were 0.30 ± 0.12, 0.59 ± 0.09, 0.79 ± 0.18 and 0.98 ± 0.08 µM for Tpm1.8, AS-Tpm1.8, Tpm1.9 and AS-Tpm1.9, respectively. The AS-extension slightly decreased the Tpm affinity to F-actin for both isoforms. The affinity of Tpm1.8 for F-actin was higher than that of Tpm1.9.

#### 2.2.2. Thermally Induced Dependences of the Light Scattering

**Figure 5:**
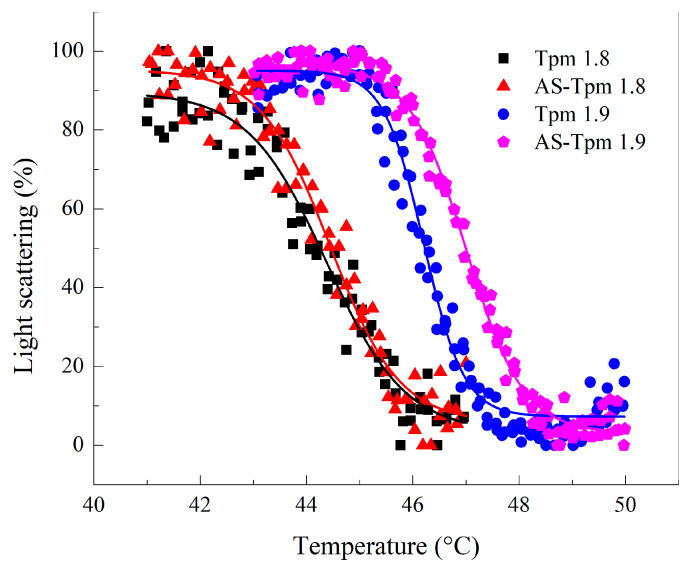
The normalized temperature dependences of the dissociation of the F-actin complexes with various Tpm isoforms. The experiments were performed in the 30 mM Hepes buffer with 100 mM NaCl; the protein concentrations were 20 µM for F-actin and 10 µM for the Tpm isoforms. The heating rate was 1 °C/min.

The stability of Tpm–F-actin complexes was estimated using the value of the temperature corresponding to the half-dissociation of Tpm from the actin surface (T_diss_). The temperature-induced dissociation curves obtained for different Tpm isoforms are presented in Figure 5. The T_diss_ values for Tpm1.8 and AS-Tpm1.8 were 44.38 ± 0.07 and 44.44 ± 0.05 °C, respectively. The stability of the Tpm1.9 and AS-Tpm1.9 complexes with F-actin was higher than that of Tpm1.8 and amounted to 46.21 ± 0.04 and 47.00 ± 0.02 °C, respectively. In the case of the Tpm1.9 isoform AS-extension slightly increased the stability of Tpm–F-actin complexes. It is worth noting that the dissociation temperatures did not correspond to any transitions in the DSC profiles.

### 2.3. Functional Properties of Tpm–F-Actin Complexes

In this section, we examined the properties of the Tpm complex with actin as a whole. The bending stiffness of the actin filament was measured, as well as the force characteristics of the myosin motors interacting with actin filaments reconstructed with different Tpm isoforms.

#### 2.3.1. Load Measurements

**Figure 6:**
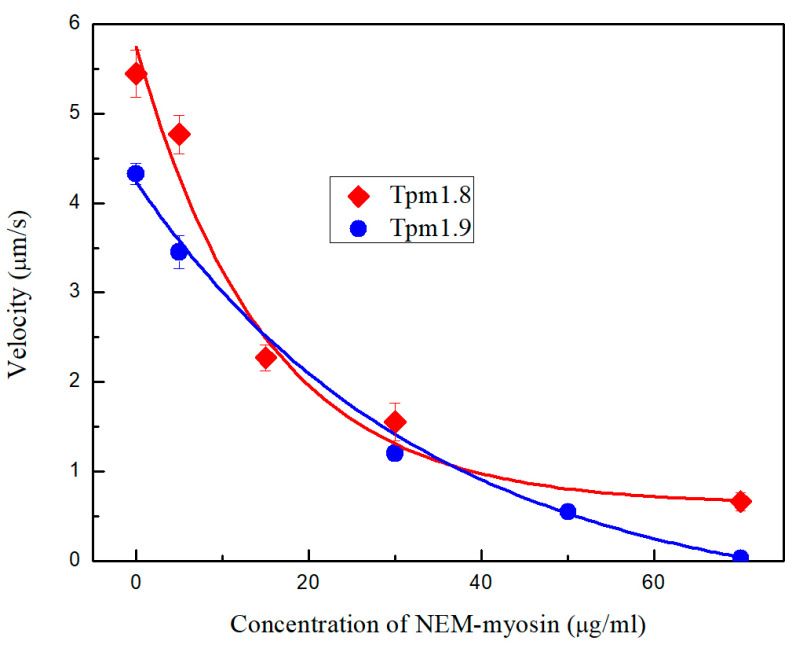
The dependence of the velocity of Tpm–F-actin complexes movement on the concentration of NEM-myosin.

In the presence of Tpm1.8 and Tpm1.9 the sliding velocity of F-actin increased from 3.6 ± 0.2 µm/s to 5.4 ± 0.3 µm/s for AS-Tpm1.8 (5.7 ± 0.1 µm/s for Tpm1.8, Figure 6) and 4.3 ± 0.1 µm/s for AS-Tpm1.9 (4.0 ± 0.2 µm/s for Tpm1.9, Figure 6), respectively.

Using the in vitro motility assay, we studied the dependence of the sliding velocity of F-actin–Tpm filaments on the NEM-myosin concentration. To decrease the sliding velocity of F-actin–Tpm filaments with Tpm1.8 and Tpm1.9 by half, 14.9 ± 3.3 µg/mL and 33.9 ± 3.2 µg/mL of NEM-myosin were required (Figure 6). Tpm1.8 complexes with actin exhibited a more cooperative velocity drop in the in vitro motility assay system than Tpm1.9 complexes. However, Tpm1.8, compared with the Tpm1.9 complexes, retained the ability to move at higher NEM-myosin concentrations.

#### 2.3.2. Bending Stiffness

To estimate the structural properties of Tpm–F-actin complexes decorated with Tpm1.8 and Tpm1.9 isoforms, we measured the bending stiffness of these complexes using a two-beam optical trap. Fibrillar actin and actin filaments decorated with Tpm were stretched in steps of 50 nm. The force and distance between the beads were registered and used for the bending stiffness calculations. The results are given in Table 2. All Tpm samples substantially increased the stiffness of F-actin. The most pronounced effect was observed for AS-Tpm1.8–F-actin complex. AS-Tpm1.8 increased the bending stiffness of the actin filaments by 3.5-fold. The deletion of the AS-extension essentially reduced the ability of the Tpm1.8 isoform to stiffen the actin filaments. Tpm1.9 and AS-Tpm1.9 equally increased the bending stiffness of F-actin by two times.

## 3. Discussion

**Figure 7:**
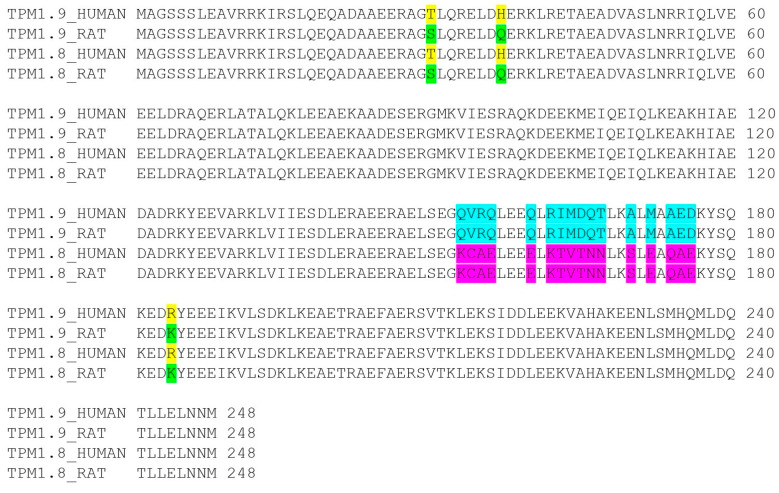
The alignment of human and rat Tpm1.8 and Tpm1.9 isoforms. Yellow and green colors highlight amino acid differences between the same human and rat isoforms. Blue and magenta colors highlight amino acid differences between the Tpm1.8 and Tpm1.9 isoforms of the same species.

In this work, we investigated the properties of two cytoplasmic Tpm isoforms, Tpm1.8 and Tpm1.9. These isoforms differ from each other only in the amino acid sequence in the central part of the Tpm molecule, corresponding to the 6th exons in their mRNA (blue and magenta, Figure 7). Despite minor differences in the primary sequence, these isoforms had significant differences in their properties. This variability in properties may have physiological significance and highlight the importance of the central part of Tpm for its functioning. In our work, we focused in detail on the biochemical and biophysical properties of these isoforms as individual molecules, as well as on the properties of their complexes formed with the filament actin, the most important partner of Tpm in cells.

### 3.1. Structural Properties of Tpm1.8 and Tpm1.9 Isoforms 

To study the structural properties of the Tpm1.8 and Tpm1.9 molecules, we used the DSC method. It can be noted that these isoforms have a common DSC profile characteristic for Tpm molecules [31,32,33]. In the profile, three domains can be distinguished, cooperatively melting in the temperature range from 30 to 70 °C. The presence of three calorimetric domains in the Tpm structure means that the molecule can be divided into three parts that melt cooperatively and independently of each other.

Calorimetric domain 3 corresponds to the *N*-terminal part of the Tpm molecule. There are several supporting facts for this statement. Firstly, the most stable domain in the DSC profiles usually corresponds to the *N*-terminal part of the Tpm molecule. Secondly, there are several studies that have shown that the protein structure corresponding to exon 1b is highly stable, indicating that this part of the molecule must melt at high temperatures [32,34,35,36]. Finally, this statement is supported by a comparison of the DSC profiles of Tpm1.6 and Tpm1.7 with Tpm1.8 and Tpm1.9, respectively. Tpm1.6 differs from Tpm1.8 only in the first exon: 1a2b is present in the structure of Tpm1.6, while 1b serves as the starting point for the synthesis of Tpm1.8. Absolutely the same differences are characteristic of the Tpm1.7 and Tpm1.9 isoforms. Thus, differences in the structures of these isoforms affect only the N-end of the molecule. Indeed, if you look at the DSC profiles for Tpm1.6 and Tpm1.7 isoforms [32], you can find that the most thermostable domain 3 differs in its melting temperature from Tpm1.8 and Tpm1.9, while other domains had similar T_m_ values. The difference in the position of the maximum of the third transition was 4.5 °C for the Tpm1.6/Tpm1.8 pair and 3.9 °C for Tpm1.7/Tpm1.9.

The least stable calorimetric domain, 1, is also very interesting. It was most logical to assume that this corresponds to the melting of the central part of the Tpm molecule, since there are many studies claiming that this part is the most flexible and unstable in Tpm [37,38,39,40,41]. This statement also agrees well with the data obtained in this work. The Tpm1.8 and Tpm1.9 isoforms differ from each other only in the 6th exon: 6b for Tpm1.8, and 6a for Tpm1.9. The largest differences in the DSC profiles of these isoforms affect calorimetric domain 1, which could logically be associated with the difference in the stability of exon 6. In total, the isoforms differ in 16 of the 248 amino acids in the central part of the molecule (Figure 7). Notably, only 5 of the 16 different amino acids had similar substitutions. Such remarkable changes indicate that the central parts of these two proteins are significantly different. It is well known that the primary sequence of Tpm is based on heptad periodicity, which determines the structural properties of their molecules. It is believed that positions 1 and 4 (**a** and **d**) in the heptad make the greatest contribution to stability; positions 5 and 7 (**e** and **g**) additionally stabilize the coiled-coil structure, while positions 2, 3, and 6 (**b**, **c,** and **f**) are mainly responsible for the interaction of Tpm with actin and partner proteins. It is noteworthy that 8 of the 16 amino acids are found in **b**, **c** and **f** positions in Tpm heptads, 5—in **a** and **d**, 3—in **e** and **g**. The **a** and **d** positions form the hydrophobic core of the Tpm molecule; therefore, charged amino acids should lead to the destabilization of the coiled-coil structure. Comparison of the central part of Tpm1.8 and Tpm1.9 primary structures showed that Tpm1.9 had charged Arg and Asp amino acids at positions 155 and 165, respectively, while Tpm1.8 had Ala and Thr at the same positions. On the contrary, Tpm1.8 had charged Glu at position 172, while Tpm1.9—Met. Both Tpm isoforms had similar charged amino acids at positions 162 and 176. Thus, both isoforms have amino acid residues that destabilize the structure, and can reduce the stability of this region, but Tpm1.9 has more charged residues in the center of the molecule, which makes domain 1 in the DSC profile for this isoform less refractory compared to Tpm1.8.

The calorimetric domain 2 corresponds to the melting of the *C*-terminal part of the Tpm molecule. This logically follows from the fact that three calorimetric domains are observed in the DSC curves, and only the *C*-terminal part of the molecule remains uncorrelated with the amino acid sequence of Tpm. Remarkably, the influence of the central part of the Tpm molecule on the protein structure is not limited to the 1st calorimetric domain. From the comparison of the DSC profiles of Tpm1.8 and Tpm1.9 (Figure 2), it is noticeable that calorimetric domain 2 which reflects the denaturation of the Tpm *C*-terminal part, is more stable in the case of the Tpm1.9 isoform than domain 2 of Tpm1.8. Based on this observation, we can conclude that the packing density of the central part of Tpm has long-range effects on its molecular structure and can affect the stability of the *C*-terminal fragment.

It is also worth noting that the stability of the rat Tpm1.8 and Tpm1.9 isoforms was studied by DSC [42]. Rat forms of Tpms differ from human ones by three amino acids, two of which are located in the *N*-terminal part of the molecule and one in the *C*-terminal part (Figure 7). All three different amino acids are located in the **b** heptad position, and two of the three substitutions are synonymous. However, there are a number of differences in the DSC profiles between the rat and human isoforms despite the general similarity in the shape of the curves. Noticeable changes affected only domain 3 of Tpm1.9: the *N*-terminal part of the human Tpm isoform had a lower melting temperature by 2.6 °C. This seems logical since two of the three replacements are located exactly in the *N*-terminal part. Moreover, as for Tpm1.8, the changes were observed both in the third and the first calorimetric domains. The effects in 3rd domain were similar to those observed for the Tpm1.9 isoform, while the melting temperature of the 1st domain for the human Tpm1.8 isoform was also 5 °C higher. This difference in the first transition is probably caused by the peculiarities of the DSC profile recording and deconvolution analysis of the rat Tpm1.8 isoform. In any case, the comparison of the human and rat isoform sequences with the DSC data shows that even small changes in the Tpm sequence can affect its structure.

An important property of Tpm molecules is their ability to form terminal interactions with neighboring molecules. Due to this feature, Tpm forms a strand on the surface of F-actin. In addition, the strength of these interactions can determine the properties of this cord. It should be noted that the solutions of both the studied Tpm isoforms have an abnormally high viscosity, which is their distinctive feature. The striated muscle Tpm1.1 isoform is perhaps the most studied Tpm isoform. The solution viscosity of Tpm1.8 measured at 200 mM NaCl is almost three times higher than that of Tpm1.1 (0.593 vs. 0.201 mPa·s), for Tpm1.9—4.5 times higher (0.886 vs. 0.201 mPa·s) [43]. The solution viscosities of these isoforms are also much higher than those obtained for the isoforms of the *TPM3* gene products (Tpm3.1, Tpm3.2. Tpm3.4, Tpm3.5, Tpm3.7) [31], as well as the Tpm4.2 isoform [33].

It is interesting to compare the obtained viscosities of Tpm1.8 and Tpm1.9 solutions with those of the Tpm1.6, Tpm1.7, and Tpm1.12 isoforms [33]. Tpm1.12, like Tpm1.8 and Tpm1.9, is a short isoform of the *TPM1* gene product that starts with exon 1b [20]. Tpm1.6 and Tpm1.7 have the same central and *C*-terminal fragment as Tpm1.8 and Tpm1.9. The viscosity values for Tpm1.8/1.9 are approximately 20–35 times higher than those for Tpm1.12. At the same time, the viscosities of the solutions of the Tpm1.6/1.7 and Tpm1.8/1.9 isoforms are comparable. This allows us to conclude that the main contributor to the strength of end-to-end interactions is the *C*-terminal part of the Tpm molecule, while the *N*-terminal part has almost no effect. It is also worth noting that at low ionic strengths, there is a significant difference in the viscosities of the Tpm1.8 and Tpm1.9 isoforms. Thus, exon 6 influences the properties of the Tpm ends. Such observations are consistent with the previously obtained data on the long-range effects of mutations in the Tpm molecule [44,45,46,47].

### 3.2. Interaction between F-Actin and Tpm1.8 and Tpm1.9

It was crucial to carry out experiments with the purpose of analyzing the interaction between Tpm and F-actin, which is the primary partner of Tpm in living cells. To examine the affinity of cytoplasmic Tpm1.8/1.9 isoforms for F-actin, a co-sedimentation assay was performed. The K_50%_ values corresponding to the Tpm concentration at which actin is half-saturated varied from 0.3 µM to 0.98 µM for all Tpm isoforms studied (Figure 4). The Tpm1.8 isoform has the highest affinity for F-actin, and the AS-Tpm1.9 isoform has the lowest affinity. The measured affinity parameters of Tpm1.8 are in good agreement with data obtained in another study [48]. In general, both studied isoforms have a high affinity to F-actin. Particular attention should be paid to the Tpm1.8 and Tpm1.12 isoforms. Their primary structure varies only in *C*-terminal exon 9, while the difference in their affinity is colossal. This can be considered as a direct evidence of the specific structure of exon 9c in Tpm1.12, which prevents the formation of contacts with the N-terminus and reduces the strength of terminal interactions between adjacent molecules and the affinity of Tpm to F-actin [33].

To study the stability of the F-actin complex with Tpm, we recorded changes in the light scattering upon heating. Usually, the stability of the complex with actin depends on the stability of the *C*-terminal part of the Tpm molecule, which is expressed by the coincidence of the melting temperatures of the 2nd calorimetric domain in the DSC profile and the dissociation temperature of the complex [31,32,33]. However, no such dependence was found for the Tpm1.8 or Tpm1.9 isoforms. The dissociation temperature of the complex, measured by the light-scattering method, is a kind of integral characteristic of stability. In other words, it reflects the impact of all the components of the interaction force simultaneously: the affinity parameter, the thermal stability of individual Tpm molecules, and the strength of end-to-end interactions. Thus, the temperature of the maximum of the 2nd transition in the DSC thermograms for Tpm1.8 or Tpm1.9 is lower than the dissociation temperature of their complexes with actin. We can conclude that the terminal interactions and high affinity of Tpm1.8/1.9 molecules make additional contribution to the stability of their complexes with F-actin.

### 3.3. Functional Peculiarities of Tpm1.8 and Tpm1.9 Complexes with Fibrillar Actin

In addition to assessing the interaction between Tpm and F-actin, it was also important to assess the properties of the complexes they form as a whole. To achieve this, we assessed the bending stiffness of actin filaments reinforced by Tpm1.8/1.9 isoforms, as well as the influence of such filaments on their interaction with myosin motors. 

The stiffness of all thin filaments is statistically different from that of F-actin (Table 2). Comparing experimental data for thin filaments with other LMW cytoplasmic Tpm isoforms and *TPM3* gene products [31], we can conclude that the Tpm1.8 and Tpm1.9 isoforms provide greater actin fiber stiffness. We can assume that such high bending stiffness parameters obtained for these isoforms may be associated with the very high affinity and strength of end-to-end interactions for the Tpm1.8 and Tpm1.9 isoforms.

Experiments with actin filaments decorated with Tpm1.8/1.9 isoforms and myosin motors also yielded a number of interesting results. Both of these isoforms led to an increase in the velocity of actin filaments along the surface coated by muscle myosin II. This indicates the ability of these Tpm isoforms to cooperatively activate the myosin motor. Moreover, a sufficiently large number of idle myosin heads is required in order to significantly inhibit the work of actin filaments decorated with these isoforms, indicating a fairly high force generated by myosin motors with Tpm1.8/1.9 isoforms.

### 3.4. Influence of Ala-Ser Extension on the Properties of Tpm1.8 and Tpm1.9 Isoforms

One of the most important post-translational modifications of Tpm is acetylation of the *N*-terminal methionine residue. The muscle Tpm1.1 isoform was shown to lose its actin filament-binding ability in the absence of acetylation [49]. It has also been shown that acetylation can influence the properties of some cytoplasmic Tpm isoforms [36,50,51,52,53,54]. In order to test how acetylation might affect the properties of Tpm1.8 and Tpm1.9 isoforms, we obtained AS-Tpm1.8/1.9 preparations with Ala-Ser extension that imitate this modification. The sufficiency of this modification in replacing the acetylation was previously shown for the muscle Tpm1.1 isoform [50]. For a long time, there was no information on the Ala-Ser modification’s effectiveness in studying the properties of non-muscle forms of Tpm. In the article by Brooker and co-authors, a comparison was made between acetylated and non-acetylated forms of Tpm, as well as AS-modified Tpm. Three types of effects were found. In the case of Tpm3.1, AS-extension did not increase the affinity of Tpm for F-actin, which was observed for acetylated Tpm3.1. In the case of Tpm1.6, this modification had identical results to the acetylated forms. In other cases, AS-extension increased the affinity of Tpm molecules for F-actin, but to a slightly lesser extent than the acetylated form [50]. Thus, AS-modification can be considered a good model for simulating acetylation, but it should be used with caution. In some situations, its effects may not be as strong as those of natural acetylation. It is worth mentioning that the primary goal of AS-modification is to eliminate the charge from *N*-terminal methionine. The results obtained using these Tpm forms are not suitable for interpreting data involving the importance of the acetyl group’s signaling function.

Upon careful analysis of the obtained results, one can notice that in most experiments, AS-modification did not have any effect on the properties of Tpm1.8/1.9. The alanine-serine terminal modification affected the viscosity of the AS-Tpm1.9 solution only at a high ionic strength of the buffer (Figure 3). The dissociation temperature of the complex with AS-Tpm1.9 was also slightly higher than of Tpm1.9 without the modification (Figure 5). The difference in the stiffness of the actin filaments with AS-Tpm1.8, and F-actin decorated by Tpm1.8, was unexpected. In all other experiments, the alanine-serine modification had no effect on the results for the Tpm1.8 isoform. The nature of this phenomenon remains unclear to us. In all likelihood, due to some undetermined factors in our experimental setup, the rigidity of the tropomyosin cord with AS-Tpm1.8 was increased. It is possible that the addition of an alanine-serine modification to the *N*-terminus of the Tpm1.8 isoform leads to a unique pattern of interaction between the Tpm strand and F-actin, which increases the integral stiffness parameter of the actin filament.

### 3.5. Possible Relationship between the Properties of Tpm1.8 and Tpm1.9 Isoforms and Their Functions in the Cell

More than 40 different tropomyosin isoforms have been identified in mammals. Such diversity contributes to the formation of actin filaments with unique properties that determine their functions in actin microcompartments and contribute to the normal existence of the cell. Studying the properties of the protein that determine the functioning of actin filaments suggests the ability to impact cellular processes that are directly related to the organization of the cytoskeleton [19]. This could be an important step towards treating many diseases. The cytoplasmic Tpm1.8 and Tpm1.9 isoforms have unique properties and form actin filaments with special physicochemical and functional features. In this section we would like to speculate a little about the influence of the Tpm1.8 and Tpm1.9 isoforms and their actin filaments on cellular functions.

High viscosity is unusual for LMW isoforms [31]. LMW isoforms are most often present in dynamic structures that require rapid filament assembly and disassembly, for which strong end-to-end interactions may be a hindrance [18,55]. Moreover, the formation of complexes between F-actin and the Tpm1.8/1.9 isoforms leads to the formation of actin filaments with high rigidity. In this sense, the presence of such strong interactions endows these isoforms with unique properties. This is in good agreement with the data obtained for Tpm1.9. Tpm1.9 is expressed in red blood cells and is present in the molecular scaffold under the membrane [23]. Experiments on mice with a deletion in exon 9d of the *TPM3* gene made it possible to obtain red blood cells with an increased content of Tpm1.9 in the framework of the red blood cell membrane [27,28]. This led to the erythrocyte F-actin becoming abnormally resistant to F-actin depolymerization. It is likely that the formation of strong interactions between Tpm and F-actin determines the participation of this type of Tpm isoform in the formation of stable actin filaments.

It is also interesting to note that the Tpm1.8/1.9 isoforms are able to suppress the metastatic phenotype [56]. Perhaps in this case, the Tpm end-binding force, or the ability to produce very stiff actin filaments, serves to slow down the rearrangement of the actin cytoskeleton, thereby stopping the division of cancer cells. The expression of Tpm1.8 and Tpm1.9 has also been shown to confer resistance to certain types of chemotherapy [57]. A probable reason for this resistance may also be associated with the formation of a stable actin cytoskeleton in the cell.

## 4. Materials and Methods

### 4.1. Protein Preparations

The Tpm isoforms used in this work were recombinant proteins. CDS for the Tpm1.8 and Tpm1.9 isoforms were obtained from the coding sequences of three Tpm isoforms (Tpm1.6, Tpm1.7, and Tpm1.12) by PCR. The primers used for cloning are presented in Table 3. In the first round of PCR, three Tpm constructs were obtained. *N*-terminal half was obtained from Tpm1.12 using primers Tpm1.12 fw and Tpm1.8/1.9 mid rev. Two *C*-terminal parts were obtained from the Tpm1.6 and Tpm1.7 coding sequences for Tpm1.8 and Tpm1.9, respectively (primers for PCR were 1.8/1.9 mid fw and Tpm1.8 rev, Tpm1.9 rev, respectively). In the second step, full coding sequences were obtained using PCR with the Tpm1.12 fw and Tpm1.8 rev and Tpm1.9 rev primers, respectively. Then DNA inserts were cloned into the pet23a+ vector between the NdeI and EcoRI restriction endonuclease sites. AS-Tpm1.8 and AS-Tpm1.9 constructs were obtained in a similar way using another PCR primer for the *N*-terminal half (AS-Tpm1.12 fw, Table 3). The correct sequence of all Tpm constructs was confirmed by sequencing at “Evrogen” (Moscow, Russia).

Tpm proteins were produced in C41 strain *E. coli* cells by 1 mM IPTG induction and overnight expression at 30 °C. After cell lysis, proteins were purified as described previously [58]. Tpm concentrations were determined spectrophotometrically using the extinction coefficient E_0.1%_ at 280 nm = 0.16 for all proteins.

G-actin was prepared from rabbit skeletal muscle acetone powder, as previously described [59]. F-actin was polymerized by the addition of 1 mM ATP, 1 mM MgCl_2,_ and 100 mM KCl to 2 mg/mL G-actin. For the thermal stability experiments with the Tpm-actin complex, F-actin was stabilized with a 1.5-fold molar excess of phalloidin. For the in vitro motility assay, F-actin was labeled with a 2-fold molar excess of TRITC-phalloidin.

### 4.2. Differential Scanning Calorimetry (DSC)

We used a MicroCal VP-Capillary differential scanning calorimeter (Malvern Instruments, Northampton, MA, USA) to perform the DSC experiments, as described earlier [32]. Briefly, all samples contained 2 mg/mL Tpm, 30 mM Hepes-Na buffer, pH 7.3 with 100 mM NaCl. The samples were heated up to 80 °C at a constant heating rate of 1 °C /min. The thermal unfolding of the Tpm species studied was fully reversible, which was verified by successive heating of the same sample. Before the DSC experiments, the Tpm1.8 and AS-Tpm1.8 samples were reduced by heating them to 60 °C for 20 min with 3 mM DTT. All Tpm samples were completely reduced after this procedure [58]. The non-two-state model [60] was used for the deconvolution analysis of the DSC profiles. Origin v.7.5 software (MicroCal Inc., Northampton, MA, USA) was used for data processing.

### 4.3. Viscosity Measurements

Viscosity measurements were performed on an AMVn falling ball micro viscometer (Anton Paar, VA, USA) using a 0.5 mL capillary, as described in [33]. DMA 4500 device (Anton Paar, VA, USA) was used to measure the specific density of the Tpm solutions and to accurately calculate the viscosity. All experiments were carried out at a Tpm concentration of 1 mg/mL in a 30 mM Hepes-Na buffer (pH 7.3) containing NaCl from 30 mM to 200 mM and 4 mM DTT. The temperature of all the solutions was 20 °C. Measurements were performed at least three times for each sample.

### 4.4. Measuring Tpm Affinity to F-Actin

The co-sedimentation assay was used to estimate the apparent affinity of Tpm species for F-actin, as previously described [43,58]. The samples contained 10 µM F-actin and Tpm at concentrations from 0.5 to 7.5 µM in 30 mM Hepes-Na buffer (pH 7.3) with 200 mM NaCl. The obtained probes were pelleted with any bound Tpm by centrifugation at 100,000× *g* (Beckman Airfuge; Beckman Coulter, Fullerton, CA, USA). SDS-PAGE was used to analyze the pellet and supernatant fractions. Protein bands were analyzed with ImageJ2 software (Scion, Frederick, MD, USA). Three independent measurements were taken for each Tpm sample.

### 4.5. Stability of Tpm–F-Actin Complexes Measured by Light Scattering

The experiments were performed at a wavelength of 350 nm on a Cary Eclipse fluorescence spectrophotometer (Varian Australia Pty Ltd., Mulgrave, VC, Australia), as described previously [58]. The samples containing 20 µM phalloidin-stabilized F-actin and 10.5 µM Tpm in a 30 mM Hepes-Na buffer (pH 7.3) with 100 mM NaCl were heated up to 65 °C at a constant heating rate of 1 °C/min. F-actin without additions was also exposed to heating under the same conditions. The temperature dependence of the light scattering for F-actin alone was deducted from the light scattering of the F-actin-Tpm complexes. The obtained dissociation curves were normalized as percentages, where the indicators of light scattering before dissociation from the actin surface were taken as 100%. Then curves were fitted by the Boltzmann sigmoidal decay function. Changes in the light scattering at 90° reflected the thermally induced disassociation of Tpm from the F-actin surface. The main parameter extracted from this analysis is *T*_diss_, i.e., the temperature at which a 50% decrease in the light scattering occurs.

### 4.6. In Vitro Motility Assay with NEM-Myosin

The effect of the Tpm isoforms on the force-generating ability of myosin was assessed by the dependence of the sliding velocity of F-actin–Tpm filaments on NEM-modified myosin added to the flow cell together with unmodified myosin [61,62]. The total amount of myosin added to the cell was 100 µg/mL. The force was expressed as a percentage of NEM-myosin required to stop F-actin–Tpm filament movement.

### 4.7. Bending Stiffness Measurements 

The method for quantifying the bending stiffness of F-actin or actin–Tpm filaments using an optical trap, as well as the mathematical model used for the data interpretation, were described earlier [31,63]. In summary, actin filaments with and without Tpm were linked to two microbeads held by a two-beam optical trap. The filament was stretched with the help of an acousto-optically controlled trap, pulling the motor bead in 50 nm steps, while the other bead was used as a force transducer. The microphotographs taken after each step were used to measure the distance between the beads and plot the force-distance curve. The experimental cell was filled by F-actin or Tpm–F-actin complex at a molar ratio of 10:1 in a buffer solution containing 25 mM KCl, 25 mM imidazole, 4 mM MgCl_2_, 1 mM EGTA, and 20 mM DTT, pH 7.5. Scavenger system with 0.2 mg/mL glucose oxidase, 0.05 mg/mL catalase, and 3 mg/mL glucose was also added to the solution.

## 5. Conclusions

Based on the results of this work, the unique characteristics of Tpm1.8 and Tpm1.9 isoforms, as well as their influence on the properties of the actin filament, were established. Both isoforms were highly stable and had an outstanding ability to form an extended strand due to end-to-end interactions. The interaction of these isoforms with actin also demonstrated very high-value parameters of affinity and stability for the formation of Tpm–F-actin complexes. Probably, the properties of these Tpm isoforms largely determine the features of the actin filament and lead to the formation of very rigid actin cables decorated with Tpm1.8 and Tpm1.9. The formation of rigid structures in vitro is in good agreement with in vivo studies. Apparently, the formation of physically stable structures correlates with the entry of such filaments into stable cellular structures.

## Figures and Tables

**Table 1:** The thermodynamic analysis of the heat absorption curves obtained by DSC.

Tpm Isoforms	T_m_ (°C)	ΔH_cal_ * (kJ∙mol^−1^)	ΔH_cal_ (% of Total)	Total ΔH_cal_ (kJ∙mol^−1^)
Tpm1.8				874
Domain 1	38.6 ± 0.09	310	35	
Domain 2	40.4 ± 0.01	332	38	
Domain 3	54.8 ± 0.08	232	27	
Tpm1.9				825
Domain 1	33.2 ± 0.10	70	8	
Domain 2	43.3 ± 0.02	640	78	
Domain 3	55.3 ± 0.05	115	14	

* The error in the given values of the calorimetric enthalpy, ΔHcal, did not exceed 10%.

**Table 2:** The parameters of bending stiffness for F-actin and Tpm–F-actin complexes.

Sample Name	Bending Stiffness, K∙10^26^N∙m^2^ (IQT, N)
F-actin	2.35 (1.6–5.3, 18)
	
F-actin + Tpm1.8	3.75 (2.7–5.55, 22) *#
F-actin + AS-Tpm1.8	8.1 (6.2–10.25, 17) *
F-actin + Tpm1.9	4.8 (3.87–6.95, 23) *#
F-actin + AS-Tpm1.9	4.7 (4–7.4, 23) *#

The difference between the stiffness of all thin filaments and F-actin is statistically significant (*). The difference between the rigidity of the thin filament and tropomyosin AS-Tpm1.8 is also statistically significant (#). Statistical differences were assessed using the nonparametric Mann–Whitney U test at a significance level of *p* < 0.05.

**Table 3:** The sequence of primers used to obtain Tpm isoforms.

Primer Name	Primer Sequence
Tpm1.12 fw	5′ATATATACATATGGCTGGAAGTAGCTCACTTGAAGC 3′
AS-Tpm1.12 fw	5′ATATATACATATGGCTAGCATGGCTGGAAGTAGCTCACTTGAAGC 3′
Tpm1.8/1.9 mid fw	5′ GAGAGAGGCATGAAAGTCATTGAGAGTCG 3′
Tpm1.8/1.9 mid rev	5′ CGACTCTCAATGACTTTCATGCCTCTCTC 3′
Tpm1.9 rev	5′ATATATAGAATTCTTACATGTTGTTCAACTCCAGTAAAGTC 3′
Tpm1.8 rev	5′ ATATATAGAATTCTTACATGTTGTTTAACTCCAGTAAAGTCTG 3′

## Data Availability

Data is contained within the article.
